# Predictors of early adulthood hypertension during adolescence: a population-based cohort study

**DOI:** 10.1186/s12889-017-4922-3

**Published:** 2017-11-28

**Authors:** Saeed Kalantari, Davood Khalili, Samaneh Asgari, Noushin Fahimfar, Farzad Hadaegh, Maryam Tohidi, Fereidoun Azizi

**Affiliations:** 1 Department of Endocrinology, Guilan University of Medical Sciences, Rasht, Iran; 2 Prevention of Metabolic Disorders Research Center, Research Institute for Endocrine Sciences, Shahid Beheshti University of Medical Sciences, Tehran, Iran; 3 Department of Biostatistics and Epidemiology, Research Institute for Endocrine Sciences, Shahid Beheshti University of Medical Sciences, Tehran, Iran; 4 Department of Epidemiology and Biostatistics, School of Public Health, Tehran University of Medical Sciences, Tehran, Iran; 5 Endocrine Research Center, Research Institute for Endocrine Sciences, Shahid Beheshti University of Medical Sciences, Tehran, Iran; 6 Present address: Prevention of Metabolic Disorders Research Center, Research Institute for Endocrine Sciences, Shahid Beheshti University of Medical Sciences, P.O. Box: 19395-4763, Tehran, Postal Code: 1985717413 Iran

**Keywords:** Hypertension, Risk factors, Adolescent, Adult, Anthropometry, Wrist, Cholesterol

## Abstract

**Background:**

Some longitudinal studies have shown that blood pressure tracks from adolescence to adulthood, yet there is limited evidence regarding the predictive factors of adulthood hypertension during adolescence. This study was conducted within the framework of the Tehran Lipid and Glucose Study (TLGS) to investigate the role of some factors in adolescence, measured in the first examination (1999–2001), to predict adulthood hypertension in the 4th examination (2009–2011).

**Methods:**

Overall, 1579 subjects, aged 10–19 years, were used for the analysis of the current study. Mean age (SD) of participants at the baseline was 14.2 (2.5) years and 55% of them were female. A forward stepwise approach (*p*-value <0.2 for enter and >0.05 for removal) was considered to keep significant covariates among common variables including gender, body mass index, waist circumference, wrist and hip circumferences, fasting blood sugar, triglycerides, high density lipoprotein cholesterol, total cholesterol (TC), systolic (SBP) and diastolic blood pressure (DBP). Variance inflation factor (VIF) showed some multicollinearity for anthropometric variables (VIFs between 3.5 and 10). Multivariable logistic regression revealed that gender, blood pressure, wrist circumference and total cholesterol in adolescents are important predictors for adulthood hypertension.

**Results:**

The risk increased by 4% and 39% per each 10 mmHg and 1 mmol/L increase in SBP/DBP and TC, respectively; additionally, females had a 70% lower risk. Among anthropometric variables, wrist circumference remained in the model, with 50% per centimeter increase in the risk of hypertension.

**Conclusions:**

Wrist circumferences and TC had significant roles in predicting hypertension through adolescence to adulthood.

**Electronic supplementary material:**

The online version of this article (10.1186/s12889-017-4922-3) contains supplementary material, which is available to authorized users.

## Background

Hypertension is a major public health threat worldwide and the leading cause of cardiovascular disease (CVD), heart failure, end-stage renal disease, and premature mortality. Atherosclerosis begins in early life and is taken into account as a pediatric problem, making identifying and management of CVD risk factors before adulthood crucial [1–3].

A systematic review of longitudinal studies has illustrated blood pressure tracking from childhood to adulthood [4]. The associations between blood pressure in early adulthood with childhood overweight and obesity, family history of hypertension, low socioeconomic status of parent and childhood blood pressure levels have been illustrated in different studies [5–11]. Adolescent obesity has also been associated with hypertension [12]. The risk estimation of hypertension is essential to implement better prevention and control programs. Hence, the determination of hypertension risk using some predictors in different age groups has been recommended [13]. Since the predicting factors of adulthood hypertension during adolescent ages are inconclusive and have not clearly defined, in the present study we evaluated the effects of adolescent anthropometric indices including body mass index (BMI), waist circumference (WC), wrist and hip circumferences along with some cardio metabolic risk factors during these ages, including fasting blood sugar (FBS), triglycerides (TG), high density lipoprotein cholesterol (HDL-C), total cholesterol (TC), Glomerular Filtration Rate (GFR) and systolic and diastolic blood pressure (SBP and DBP, respectively), on the development of adult hypertension.

## Methods

### Study population

Tehran Lipid and Glucose Study (TLGS) is community-based prospective study being performed on a representative sample of an urban Iranian population from Tehran. The study was initiated in 1999–2001 to estimate the prevalence of non-communicable disease risk factors. The study is ongoing and designed to continue and follow the participants for at least 20 years on triennial basis. Rationale and study design of TLGS has been discussed elsewhere [14, 15].

Participants of the TLGS who attended both the first (1999–2001) and 4th (2008–2011) examinations were eligible for the present study. Of a total 2954 individuals, aged 10–19 years, 170 were excluded due to missing data at first examination. After excluding participants with missing data on SBP and DBP at 4th examination or lost to follow-up data (*n* = 1205), 1579 subjects were considered for the current study. None of the selected subjects had any kind of cancer and 41 had non-ischemic cardiovascular disease who were not excluded. Interval time from adolescence to adulthood (from 1st exam to the 4th one in the TLGS) had a median of 10.5 years with an interquartile range of 9.5–11.3 years.

The Ethics Committee of the Research Institute for Endocrine Sciences, Shahid Beheshti University of Medical Sciences approved the design of the TLGS study, and all participants provided with written informed consent.

### Measurements

Trained medical doctors and nurses collected the information and samples according to the standard methods [14–16]. Using digital scale (model 707, range 0.1–150 kg, Seca, Hamburg, Germany), weight was measured in daylight when scale was placed on a flat surface and calibrated to zero before measurement and participant had not shoes and minimally clothed; finally the value was recorded to the nearest 100 g. Without shoes and in a standing position, height was measured using tape meter (model 208 Portable Body Meter Measuring Devise; Seca), whilst the shoulders were in a normal situation. Participants’ BMI (Kg/m2) was calculated; and using un-stretched tape meter and over light clothing, waist and hip circumferences were measured at the umbilical level and at the widest girth of the hip respectively, without any pressure to body surface, and recorded to the nearest 1 cm. To measure wrist circumference subjects held their anterior wrist surface upward, without any tape-measure pressure over it; the superior border of the tape was located just distal to the prominences of radial and ulnar bones and circumference was measured to the nearest 0.1 cm [17–19]. To avoid subjective error, all measurements were taken by the same person. Quality control mechanisms had been developed for all measurements in TLGS to assure that data were collecting uniformly over time. The process was adopted from “ARIC” study [14–16].

Using a standard mercury sphygmomanometer calibrated by Iranian Institute of Standards and Industrial Researches, blood pressure was measured on the right arm at the heart level twice with at least a 30-s interval [20, 21], while participants were in a relaxed sitting position. The values were recorded in millimeters of mercury. Concerning the circumference of the participant’s arm a pediatric, regular adult, or large cuff were chosen (if appropriate). A blood sample was drawn between 7:00 & 9:00 am after 12–14 hs overnight fasting. Samples were centrifuged within 30–45 min of collection. All laboratory analyses were performed at TLGS laboratory on the day of blood collection. Serum glucose, TC, TG and HDL-C were measured by the enzymatic colorimetric method, using commercial kits (Pars Azmoon, Tehran, Iran) and a Selectra II auto analyzer (Vital Scientific, Spankeren, Netherlands). For glucose, glucose oxidase, for TC cholesterol esterase and cholesterol oxidase, and for TG, glycerol phosphate oxidase was used. HDL-C was assayed after precipitation of the apolipoprotein B containing lipoproteins with phosphotungstic acid. To monitor the quality of laboratory measurements, assayed serum controls at two different concentrations were used. Intra- and inter-assay coefficients of variation (CVs) were both <2.2% for glucose, 1.8% for TC, 1.9% for TG and 2.9% for HDL-C at both baseline and follow-up examinations.

Serum creatinine was measured using the photometric Jaffe method (Pars Azmoon Kit, Tehran, Iran); intra-assay CVs were 1.7%. Estimated glomerular filtration rate (eGFR) was calculated by using equations of Schwartz [22, 23].

The outcome, adult hypertension, was defined as SBP >140 mmHg, DBP >90 mmHg, or taking antihypertensive medications.

Hypertension in adolescent was defined as age, gender and height specific blood pressure that was equal or greater than 95th percentile [24, 25].

### Statistical analysis

All continuous data with normal distribution are shown as mean (SD), skewed variables as median, inter-quartile (IQ range), and categorical variables are expressed as frequency (percentage). A t-test was used to evaluate the difference between continuous variables. Difference for categorical variables was assessed using Chi square test. The Mann-Whitney test was applied for TG as a not normally distributed variable.

Scatter plot with Loess-fitted line was used to show the relationship between anthropometric measures at adolescence with systolic and diastolic blood pressures at adulthood. Logistic regression analysis was used to study the effect of independent variables during adolescence on hypertension in adulthood. Multicollinearity of independent variables was checked via the variance inflation factor (VIF) statistic. A forward stepwise approach was considered to keep significant covariates among age, gender, BMI, waist, wrist, height and hip circumference, FBS, TG, HDL-C, TC, eGFR, SBP and DBP. To be more conservative for negative confounding and missing effective variables we considered a *p*-value of <0.2 for entry and a p-value of >0.05 for removal. The Area Under the Curve (AUC) and Hosmer-Lemeshow goodness of fit test were considered as discrimination and calibration indices of the model.

We calculated the probability of being lost to follow-up for each subject based on his/her baseline characteristics; it is a kind of propensity score (PS) estimated by logistic regression model [26]. To correct the lost to follow-up bias, we forced the PS into the model as a covariate. We also forced age at 4th examination into the model.

All interactions between sex and potential risk factors were checked; there was no significant interaction and a pooled gender analysis was implemented to improve the power.

All analysis was executed using SPSS 20 statistical software.

## Results

Mean (±SD) age of participants at baseline was 14 (±2.53) years and 55% of them were female. Included and excluded participants (for missing data) had no differences in characteristic at baseline, except for clinically negligible, albeit statistically significant, differences in gender, TG and eGFR variables (Table 1). Baseline characteristics of adolescent participants according to their adulthood hypertension are shown in Table 2.

**Table 1:** Baseline characteristics of the TLGS adolescents among included and excluded participants

	Excluded participants (*N* = 1375)	Included participants (*N* = 1579)	*P*-value
Age, y	14.03(2.53)	14.16(2.50)	0.16
Female, n (%)	657(47.8)	874(55.4)	<0.001
Body Mass Index, kg/m^2^	19.85(4.40)	20.0(4.22)	0.36
Waist circumference, cm	68.74(11.05)	68.60(10.28)	0.73
Wrist circumference, cm	15.30(1.43)	15.27(1.38)	0.78
Hip circumference, cm	85.47(11.97)	86.22(11.75)	0.09
Systolic blood pressure, mm Hg	105.3(12.0)	105.0(11.6)	0.42
Diastolic blood pressure, mm Hg	71.3(9.3)	70.8(9.1)	0.15
Fasting blood glucose, mmol/L	4.93(0.68)	4.90(0.44)	0.19
Total cholesterol, mmol/L	4.34(0.86)	4.37(0.83)	0.43
Triglyceride^a^, mmol/L	1.05(0.70)	1.09(0.70)	0.01
High density lipoprotein, mmol/L	1.12(0.27)	1.12(0.28)	0.93
Glumerolar Filteration Rate ml/min/1.73 m^2^	111.37(16.0)	110.11(15.78)	0.03

Mean (SD) shown for continuous variables and P value calculated with t-test; % shown for categorical variables with *P* value according to chi-square

^a^Triglyceride was shown as median (inter-quartile range) and *P* value according to Mann-Whitney test

**Table 2:** Baseline characteristics of the TLGS adolescents (1999–2001) by their hypertension occurrence in adulthood (2008–2011)

	Non- hypertension (*N* = 1514)	Hypertension (*N* = 65)	*P*-value	VIF^a^
Age, y	14.12(2.50)	15.11(2.26)	0.002	2.24
Female, n (%)	862(56.9)	12(18.5)	<0.001	2.85
Body Mass Index, kg/m^2^	19.83(4.04)	24.05(5.96)	<0.001	7.20
Waist circumference, cm	68.16(10.21)	78.85(14.40)	<0.001	5.57
Wrist circumference, cm	15.21(1.34)	16.72(1.43)	<0.001	3.48
Hip circumference, cm	85.90(11.62)	93.85(12.17)	<0.001	9.29
Systolic blood pressure, mm Hg	104.5(11.3)	115.7(13.7)	<0.001	1.80
Diastolic blood pressure, mm Hg	70.6(9.1)	76.6(9.2)	<0.001	1.54
Fasting blood glucose, mmol/L	4.90(0.44)	4.90(0.47)	0.74	1.05
Total cholesterol, mmol/L	4.35(0.81)	4.70(1.06)	0.002	1.42
Triglycerides^a^, mmol/L	1.10(0.70)	1.19(0.86)	0.04	1.30
High density lipoprotein, mmol/L	1.12(0.27)	1.09(0.26)	0.34	1.35
Glumerolar Filteration Rate ml/min/1.73 m2	109.96(15.78)	113.54(15.64)	0.80	1.75
Adolescent hypertension, yes (%)	78(5.5)	11(16.9)	<0.001	1.23

Mean (SD) shown for continuous variables and P value calculated with t-test; % shown for categorical variables with P value according to chi-square; TG was shown as median (inter-quartile range) and P value according to Mann-Whitney test

^a^VIF when all covariates are in the model

It was ascertained that during 10 years of follow up, 65 out of 1579 adolescents developed hypertension in their adulthood, indicating a cumulative incidence of 0.04 (95% CI: 0.03–0.05). Of these, 5 (7.7%) were using hypertension drugs and others (92.3%) had systolic and/or diastolic hypertension as unknown hypertensive subjects.

Individuals with early adulthood hypertension were one year older at baseline with higher BMI, WC, wrist and hip circumference. They also had higher level of SBP, DBP, TGs and TC; VIF values showed low level of co-linearity for most independent variables, except for the anthropometric ones, in which the range of VIFs was 3.5 to 10.

Multivariable logistic regression with forward stepwise method revealed that among independent variables, the most important ones with significant effects on early adulthood hypertension were gender, adolescent blood pressure, wrist circumference and total cholesterol. Females had a 70% lower risk; each 10 mmHg increase in systolic and diastolic blood pressure increased the risk by 4 and 3% respectively, and each 1 mmol/L increase in TC increased the risk of hypertension by 39%. Among anthropometric variables, only wrist circumference with 50% per centimeter increase in risk of hypertension remained in the model (Table 3). An AUC of 0.84 (95% CI: 0.79–0.89) and a Hosmer-Lemeshow chi^2^ of 4.3 (*p*-value = 0.83) showed a good performance of the model.

**Table 3:** Odds Ratios (95% CIs) of significant variables in adolescence for hypertension events in adulthood

Independent variable	OR (95% CI)	*P*-value
Model 1		
Age at 4th exam^a^, years	1.02(0.91–1.15)	0.69
Gender, female	0.31(0.15–0.64)	0.001
Systolic blood pressure, 10 mmHg	1.03(1.01–1.06)	0.02
Diastolic blood pressure, 10 mmHg	1.04(1.01–1.08)	0.02
Wrist, cm	1.50(1.18–1.89)	0.001
Total cholesterol, mmol/L	1.39(1.06–1.84)	0.02
Propensity score^a^, %	1.01(0.99–1.03)	0.15

A forward stepwise approach was considered to keep significant covariates among gender, BMI, waist, wrist and hip circumference, FBS, TG, HDL-C, TC, eGFR, SBP and DBP with a p-value of <0.2 for enter and >0.05 for removal

The AUC of the model was 0.84 (95% CI: 0.79–89) and its Hosmer-Lemeshow chi^2^ was 4.3 (*p* = 0.83)

^a^Age at 4th examination and propensity score for follow-up was forced into the model

**Fig. 1:**
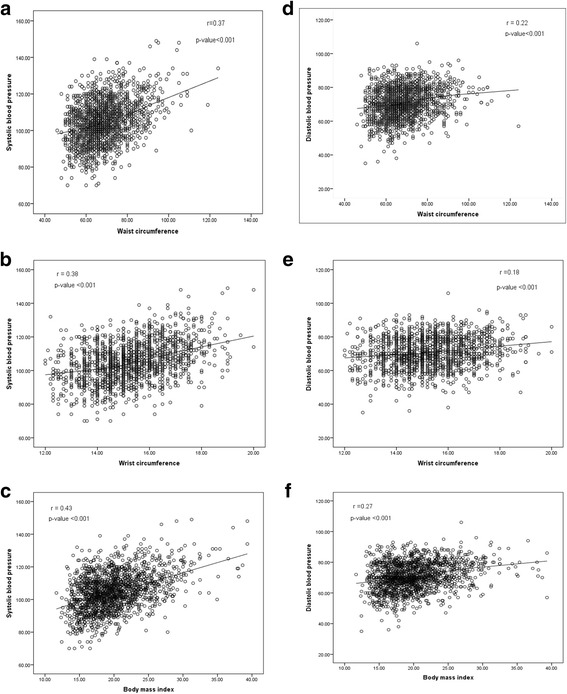
Scatter plot between systolic blood pressure and BMI (**a**), waist circumference (**b**), wrist circumference (**c**) and diastolic blood pressure and BMI (**d**), waist circumference (**e**), wrist circumference (**f**) along with a line of best fit (Loess methods)

Figure 1 shows scatter plots for association of systolic and diastolic blood pressure in early adulthood with waist, wrist and BMI in adolescence. The line of best fit (Loess methods) shows linear association between anthropometrics and blood pressure, with the association between SBP and wrist circumference being more obvious (less spheroid pattern and significant slip, Pearson’s correlation coefficient of 0.38, *p* < 0.001).

## Discussion

Hypertension is a crucial modifiable risk factor of mortality and morbidity originating in childhood [27]. Early identification of the population at risk, would make it possible to identify and modulate the risk factors and underlying causes. In this study we evaluated several potential adolescent risk factors for adult hypertension in a cohort of adolescents followed for about 10 years. To the best of our knowledge, this is the first study that has introduced serum TC and wrist circumferences as risk factors of hypertension in early adulthood.

The association of childhood adiposity with several CVD risks like hypertension, hyperlipidemia and diabetes mellitus in adulthood has been reported in many studies [5, 7–10, 28–32].

In the Bogalusa [7], Muscatine [8] and YFS [10] cohorts, childhood adiposity was a predictor of diabetes and low HDL. In the Bogalusa and CDAH [9] cohorts, an association was observed between BMI in childhood and Low Density Lipoprotein (LDL) levels; in all of these large studies there was a significant correlation between hypertension and triglyceride levels. In our cohort study, serum TC and TG concentrations during adolescence were significantly correlated with adulthood hypertension in univariable analysis (*P* < 0.05); an association however was not demonstrated for FBS and HDL-C. Our results only showed a significant correlation between adolescent TC and adulthood hypertension using multivariable analysis. It was demonstrated that for each 1 mmol/L increase in TC during adolescence the risk of adult hypertension increased by 39%.

The relationship between elevated blood pressure and weight begins in early childhood. Obesity can progressively raise blood pressure by altering cardiac output, cardiac systolic and diastolic function, renal pressure natriuresis [33] and sympathetic nervous system and renin-angiotensin-aldosterone system activation [34]. In a retrospective study of a large cohort of subjects aged between 2 and 29 years, systolic and diastolic blood pressure increased with increasing BMI in all age groups [35]. In another study conducted on 530 children from birth to three years of age, increasing weight in the first six months of life were associated with higher systolic blood pressure at three years of life [36]. Contrary to these findings, a report from the Bogalusa Heart study on a cohort of 11,478 children revealed that mean systolic and diastolic blood pressure levels did not increase despite a rise in the prevalence of obesity from 6 to 17% over the 19 year follow up period [37].

Hu et al. [38] reported that increased BMI and WC and their combination in particular were directly associated with high blood pressure in 1145 Chinese school children, aged 7–17 years. Flodmark et al. [39] in their survey of a sample of obese Swedish children showed that WC was significantly correlated with atherogenic risk factors. In another large scale study conducted on 78,000 children in China, BMI and WC were positively correlated with SBP and DBP, and central obesity was found to have a stronger association with the risk of hypertension than BMI [40].

The present study consistent with data of others, reported a linear correlation between SBP and DBP in early adulthood with WC and BMI in adolescents; these anthropometric indexes were higher significantly in hypertensive compared to normotensive adults [33, 34, 41]. Due to substantial collinearity among anthropometric measurements, these two measurements, along with hip circumference, did not remain in the model in the presence of wrist circumference, indicating the importance of wrist in the prediction of hypertension compared to the other measures. However, we repeated the analysis for each anthropometric variable separately and compared the discrimination and calibration of the models. The results were the same for discrimination but the calibration was better for the model including wrist circumference; in the models including other anthropometrics, total cholesterol did not remain in the model (Additional file 1: Tables S1-S3). Since the multicollinearity for wrist was less than that for other anthropometrics, we also considered wrist with one of the other anthropometrics each time; still wrist remained in the model and the other anthropometric was removed by stepwise method.

Wrist circumference has not been measured and considered as a predictor of adulthood hypertension in any study ever published. Our findings indicated that wrist circumference in adolescence had a linear correlation with DBP and SBP in adulthood. Capizzi et al. [42] in a study conducted on 477 overweight/obese children and adolescents (mean age 10.3) detected a close relationship among wrist circumference, its bone components, and insulin resistance in overweight/obese children and adolescents, suggesting new perspectives for CVD prediction and an easy to measure clinical marker of insulin resistance, which can be used to identify young individuals at high risk for cardiovascular diseases.

Similar results have been recently documented in adults as well; in a cohort study of 3642 adult women, it was shown that wrist is an independent risk factor of incident hypertension and CVD events [43]; Amini et al. reported wrist circumference as a clinical marker to identify individuals at risk of cardiometabolic disorders [44]; Jahangiri-Noudeh et al., in a population aged ≥20 years, detected a predictor role of wrist circumference for future development of diabetes in both genders, a finding that remained significant even after controlling for BMI or WC among females [18]. Additionally, wrist circumference, considered as a measure of peripheral fat distribution and skeletal frame size in children [45, 46] was not severely confounded by body fat variation [47].

Childhood blood pressure has been reported as a predictor of adulthood hypertension in many studies [5, 29, 41, 48, 49]. In agreement with current data, we also showed that the magnitude of blood pressure in adolescents predicts future hypertension in adulthood. We demonstrated that for each 10 mmHg increase in systolic or diastolic childhood blood pressure the risk of adult hypertension increased by 3% in our study group.

Gender has also been reported to be associated with hypertension in childhood and in later life by some studies; a Canadian study on a cohort of 1267 adolescents demonstrated a greater prevalence of higher SBP in boys than girls as they approach adulthood [50]. Field et al. [32] conducted a prospective study on 314 children with an age range of 8–15 years at baseline and followed them for 8–12 years, incidence of elevated blood pressure was higher among male subjects (12.3% vs. 1.9%); in line with these studies, we also reported adolescent females as having a 70% lower risk for development of SBP and DBP.

Since some studies have demonstrated the effect of adolescent’s height on blood pressure [24, 25], in a further analysis we adjusted it in the model but the results did not change (data not shown). At baseline, 89 adolescents had age- gender- and height-specific hypertension that none of them used any medication. Considering the difference in the definition of hypertension in adolescence and adults, we could not exclude those adolescence from our analyses. Moreover, only 11 of those 89 subjects (12%) had developed early adulthood hypertension and adjustment for adolescent hypertension in the final model did not change the results (data not shown). Consequently, the effect of blood pressure in adolescence on hypertension in adulthood was determined without any restriction in all ranges of blood pressure at baseline.

To mention the strengths and limitations of our study, regarding the latter, we may not be able to generalize our findings to other populations and propose that our results hence be appraised in other countries in different ethnic groups. Although family history of hypertension and socioeconomic status have been cited to have association with adulthood hypertension, as a limitation of our study we did not have a reliable report in this regard. We also did not have any data regarding dietary habit, however it’s intermediate variables including BMI, WC, FBS, TG, TC and blood pressure have been included in the analyses. As strengths, we followed a large cohort of adolescents for 10 years to determine the predictive value of some variables for the incidence of hypertension in their adulthood. Although a part of our participants have been excluded because of missing data or being lost to follow-up, there was no clinically important difference between included and excluded subjects regarding studied variables at baseline.

## Conclusions

Regarding the importance of early identification of at risk population, we found that wrist circumference, as a simple and easy-measurable anthropometric index, besides adolescent total cholesterol and blood pressure, can be used as predictors of adulthood hypertension in adolescents.

## Additional file

Additional file 1: Table S1. Odds Ratios (95% CIs) of significant variables in adolescence for hypertension events in adulthood, considering BMI as anthropometric variable. **Table S2.** Odds Ratios (95% CIs) of significant variables in adolescence for hypertension events in adulthood, considering waist circumference as anthropometric variable. **Table S3.** Odds Ratios (95% CIs) of significant variables in adolescence for hypertension events in adulthood, considering hip circumference as anthropometric variable. (DOCX 16 kb)

## Abbreviations

BMI: Body mass index
CVD: Cardiovascular disease
DBP: Diastolic blood pressure
eGFR: Estimated glomerular filtration rate
FBS: Fasting blood sugar
HDL-C: High density lipoprotein cholesterol
LDL: Low Density Lipoprotein
SBP: Systolic blood pressure
TC: Total cholesterol
TG: Triglycerides
TLGS: Tehran Lipid and Glucose Study
VIF: Variance inflation factor
WC: Waist circumference

## References

[1] Kim NY, Hong YM, Jung JW, Kim NS, Noh CI, Song Y-H (2013). The relationships of body mass index, waist-to-height ratio, and body fat percentage with blood pressure and its hemodynamic determinants in Korean adolescents: a school-based study. Korean J Pediatr.

[2] McGill HC, McMahan CA, Gidding SS (2008). Preventing heart disease in the 21st century implications of the pathobiological determinants of atherosclerosis in youth (PDAY) study. Circulation.

[3] Mohammadi SG, Mirmiran P, Bahadoran Z, Mehrabi Y, Azizi F (2015). The Association of Dairy Intake with Metabolic Syndrome and its Components in adolescents: Tehran lipid and glucose study. Int J Endocrinol Metab.

[4] Chen X, Wang Y (2008). Tracking of blood pressure from childhood to adulthood a systematic review and meta–regression analysis. Circulation.

[5] Juhola J, Oikonen M, Magnussen CG, Mikkilä V, Siitonen N, Jokinen E (2012). Childhood physical, environmental and genetic predictors of adult hypertension: the cardiovascular risk in young Finns study. Circulation.

[6] Sun SS, Grave GD, Siervogel RM, Pickoff AA, Arslanian SS, Daniels SR (2007). Systolic blood pressure in childhood predicts hypertension and metabolic syndrome later in life. Pediatrics.

[7] Berenson GS, Srinivasan SR, Bao W, Newman WP, Tracy RE, Wattigney WA (1998). Association between multiple cardiovascular risk factors and atherosclerosis in children and young adults. N Engl J Med.

[8] Davis PH, Dawson JD, Riley WA, Lauer RM (2001). Carotid intimal-medial thickness is related to cardiovascular risk factors measured from childhood through middle age the muscatine study. Circulation.

[9] Magnussen CG, Raitakari OT, Thomson R, Juonala M, Patel DA, Viikari JS (2008). Utility of currently recommended pediatric Dyslipidemia classifications in predicting Dyslipidemia in adulthood evidence from the childhood determinants of adult health (CDAH) study, cardiovascular risk in young Finns study, and Bogalusa heart study. Circulation.

[10] Raitakari OT, Juonala M, Kähönen M, Taittonen L, Laitinen T, Mäki-Torkko N (2003). Cardiovascular risk factors in childhood and carotid artery intima-media thickness in adulthood: the cardiovascular risk in young Finns study. JAMA.

[11] Kuczmarski RJ, Ogden CL, Guo SS, Grummer-Strawn LM, Flegal KM, Mei Z (2002). 2000 CDC growth charts for the United States: methods and development. Vital and health statistics 11.

[12] Movahed MR, Bates S, Strootman D, Sattur S (2011). Obesity in adolescence is associated with left ventricular hypertrophy and hypertension. Echocardiography.

[13] Lackland DT (2010). Hypertension risk prediction an important but complicated assessment. Hypertension.

[14] Azizi F, Ghanbarian A, Momenan AA, Hadaegh F, Mirmiran P, Hedayati M (2009). Prevention of non-communicable disease in a population in nutrition transition: Tehran lipid and glucose study phase II. Trials.

[15] Azizi F, Rahmani M, Emami H, Mirmiran P, Hajipour R, Madjid M (2002). Cardiovascular risk factors in an Iranian urban population: Tehran lipid and glucose study (phase 1). Soz Praventivmed.

[16] Azizi F, Madjid M, Rahmani M, Emami H, Mirmiran P, Hadjipour R (2000). Tehran lipid and glucose study (TLGS): rationale and design. Iran J Endocrinol Metab.

[17] Hadaegh F, Zabetian A, Harati H, Azizi F (2006). Waist/height ratio as a better predictor of type 2 diabetes compared to body mass index in Tehranian adult men-a 3.6-year prospective study. Exp Clin Endocrinol Diabetes.

[18] Jahangiri Noudeh Y, Hadaegh F, Vatankhah N, Momenan AA, Saadat N, Khalili D (2013). Wrist circumference as a novel predictor of diabetes and prediabetes: results of cross-sectional and 8.8-year follow-up studies. J Clin Endocrinol Metab.

[19] Mirmiran P, Mohammadi F, Allahverdian S, Azizi F (2002). Association of educational level and marital status with dietary intake and cardiovascular risk factors in Tehranian adults: Tehran lipid and glucose study (TLGS). Nutr Res.

[20] Perloff D, Grim C, Flack J, Frohlich ED, Hill M, McDonald M (1993). Human blood pressure determination by sphygmomanometry. Circulation.

[21] McAlister FA, Straus SE (2001). Measurement of blood presssure: an evidence based review. BMJ.

[22] Ghasemi A, Azimzadeh I, Afghan M, Momenan A, Bagheripour F, Azizi F (2015). Pediatric reference values for serum Creatinine and estimated Glomerular filtration rate in Iranians: Tehran lipid and glucose study. Arch Iran Med.

[23] Schwartz G, Brion L, Spitzer A (1987). The use of plasma creatinine concentration for estimating glomerular filtration rate in infants, children, and adolescents. Pediatr Clin N Am.

[24] Rosner B, Prineas RJ, Loggie JM, Daniels SR (1993). Blood pressure nomograms for children and adolescents, by height, sex, and age, in the United States. J Pediatr.

[25] Voors AW, Webber LS, Frerichs RR, Berenson GS (1977). Body height and body mass as determinants of basal blood pressure in children--the Bogalusa heart study. Am J Epidemiol.

[26] D'Agostino RB (1998). Propensity score methods for bias reduction in the comparison of a treatment to a non-randomized control group. Stat Med.

[27] Chobanian AV, Bakris GL, Black HR, Cushman WC, Green LA, Izzo JL (2003). The seventh report of the joint national committee on prevention, detection, evaluation, and treatment of high blood pressure: the JNC 7 report. JAMA.

[28] Yoon KL (2013). Does hypertension begin in adolescence?. Korean J Pediatr.

[29] Israeli E, Ze K, Tekes-Manova D, Tirosh A, Schochat T, Bernheim J (2007). Blood-pressure categories in adolescence predict development of hypertension in accordance with the European guidelines. Am J Hypertens.

[30] Juonala M, Viikari JS, Raitakari OT (2013). Main findings from the prospective cardiovascular risk in young Finns study. Curr Opin Lipidol.

[31] Juonala M, Magnussen CG, Berenson GS, Venn A, Burns TL, Sabin MA (2011). Childhood adiposity, adult adiposity, and cardiovascular risk factors. N Engl J Med.

[32] Field AE, Cook NR, Gillman MW (2005). Weight status in childhood as a predictor of becoming overweight or hypertensive in early adulthood. Obes Res.

[33] Hall JE, Brands MW, Henegar JR (1999). Mechanisms of hypertension and kidney disease in obesity. Ann N Y Acad Sci.

[34] Rahmouni K, Correia ML, Haynes WG, Mark AL (2005). Obesity-associated hypertension new insights into mechanisms. Hypertension.

[35] Falkner B, Gidding SS, Ramirez-Garnica G, Wiltrout SA, West D, Rappaport EB (2006). The relationship of body mass index and blood pressure in primary care pediatric patients. J Pediatr.

[36] Belfort MB, Rifas-Shiman SL, Rich-Edwards J, Kleinman KP, Gillman MW (2007). Size at birth, infant growth, and blood pressure at three years of age. J Pediatr.

[37] Freedman DS, Goodman A, Contreras OA, DasMahapatra P, Srinivasan SR, Berenson GS (2012). Secular trends in BMI and blood pressure among children and adolescents: the Bogalusa heart study. Pediatrics.

[38] Hu Y, Reilly K, Liang Y, Xi B, Liu J, Xu D (2011). Increase in body mass index, waist circumference and waist-to-height ratio is associated with high blood pressure in children and adolescents in China. J Int Med Res.

[39] Flodmark C-E, Sveger T, Nilsson-Ehle P (1994). Waist measurement correlates to a potentially atherogenic lipoprotein profile in obese 12–14–year-old children. Acta Paediatr.

[40] Lu X, Shi P, Luo C-Y, Zhou Y-F, H-T Y, Guo C-Y (2013). Prevalence of hypertension in overweight and obese children from a large school-based population in Shanghai, China. BMC Public Health.

[41] Tirosh A, Afek A, Rudich A, Percik R, Gordon B, Ayalon N (2010). Progression of normotensive adolescents to hypertensive adults a study of 26 980 teenagers. Hypertension.

[42] Capizzi M, Leto G, Petrone A, Zampetti S, Papa RE, Osimani M (2011). Wrist circumference is a clinical marker of insulin resistance in overweight and obese children and adolescents. Circulation.

[43] Mohebi R, Mohebi A, Sheikholeslami F, Azizi F, Hadaegh F (2014). Wrist circumference as a novel predictor of hypertension and cardiovascular disease: results of a decade follow up in a west Asian cohort. J Am Soc Hypertens.

[44] Amini A, Soltanian N, Iraj B, Askari G, Ebneyamin S, Ghias M (2012). Association of wrist circumference with cardio metabolic risk factors. J Pak Med Assoc.

[45] Grant J, Custer P, Thurlow J (1981). Current techniques of nutritional assessment. Surg Clin North Am.

[46] Miller JZ, Slemenda CW, Meaney FJ, Reister TK, Hui S, Johnston CC (1991). The relationship of bone mineral density and anthropometric variables in healthy male and female children. Bone Miner.

[47] Ferrante E, Pitzalis G, Deganello F, Galastri E, Sciarpelletti R, Imperato C (1992). The evaluation of body composition in children by anthropometry and impedance measurement. Minerva Pediatr.

[48] Charan J, Buch N, Goyal JP, Kumar N, Parmar I, Shah VB (2011). Prevalence of hypertension in school going children of Surat city, western India. J Cardiovasc Dis Res.

[49] Petkeviciene J, Klumbiene J, Simonyte S, Ceponiene I, Jureniene K, Kriaucioniene V (2014). Physical, Behavioural and genetic predictors of adult hypertension: the findings of the Kaunas cardiovascular risk cohort study. PLoS One.

[50] Dasgupta K, O’Loughlin J, Chen S, Karp I, Paradis G, Tremblay J (2006). Emergence of sex differences in prevalence of high systolic blood pressure analysis of a longitudinal adolescent cohort. Circulation.

