# Supplementary material for: Predictors of early adulthood hypertension during adolescence: a population-based cohort study
Source: BMC Public Health. 2017 Nov 28;17:915. doi: 10.1186/s12889-017-4922-3 (PMC5706303; doi:10.1186/s12889-017-4922-3)
Supplement: Additional file 1: Table S1. — Odds Ratios (95% CIs) of significant variables in adolescence for hypertension events in adulthood, considering BMI as anthropometric variable. Table S2. Odds Ratios (95% CIs) of significant variables in adolescence for hypertension events in adulthood, considering waist circumference as anthropometric variable. Table S3. Odds Ratios (95% CIs) of significant variables in adolescence for hypertension events in adulthood, considering hip circumference as anthropometric variable. (DOCX 16 kb) [file 12889_2017_4922_MOESM1_ESM.docx]

**Table S1: Odds Ratios (95% CIs) of significant variables in adolescence for hypertension events in adulthood, considering BMI as anthropometric variable**

| **Independent variable** | **OR (95% CI)** | **P-value** |
| --- | --- | --- |
| **Model 1** |  |  |
| Age at 4^th^ exam*, years | 1.06(0.95-1.19) | 0.29 |
| Gender, female | 0.18(0.09-0.36) | 0.001 |
| Systolic blood pressure, cm Hg | 1.03(1.00-1.06) | 0.05 |
| Diastolic blood pressure, cm Hg | 1.04(1.01-1.08) | 0.02 |
| BMI, kg/m2 | 1.12(1.06-1.18) | 0.001 |
| Propensity score*, % | 1.01(0.99-1.03) | 0.15 |

A forward stepwise approach was considered to keep significant covariates among gender, BMI, , FBS, TG, HDL-C, TC, eGFR, SBP and DBP with a p-value of <0.2 for enter and >0.05 for removal.

The AUC of the model was 0.84 (95% CI: 0.79-0.89) and its Hosmer-Lemeshow chi^2^ was 13.1 (p=0.11).

*Age at 4^th^ examination and propensity score for follow-up was forced into the model.

**Table S2: Odds Ratios (95% CIs) of significant variables in adolescence for hypertension events in adulthood, considering waist circumference as anthropometric variable**

| **Independent variable** | **OR (95% CI)** | **P-value** |
| --- | --- | --- |
| **Model 1** |  |  |
| Age at 4^th^ exam*, years | 1.06(0.95-1.18) | 0.32 |
| Gender, female | 0.20(0.10-0.39) | 0.001 |
| Systolic blood pressure, cm Hg | 1.03(1.00-1.06) | 0.04 |
| Diastolic blood pressure, cm Hg | 1.05(1.01-1.08) | 0.01 |
| Waist circumference, cm | 1.05(1.02-1.07) | 0.001 |
| Propensity score*, % | 1.01(0.99-1.03) | 0.21 |

A forward stepwise approach was considered to keep significant covariates among gender, waist circumference, FBS, TG, HDL-C, TC, eGFR, SBP and DBP with a p-value of <0.2 for enter and >0.05 for removal.

The AUC of the model was 0.84 (95% CI: 0.79-0.89) and its Hosmer-Lemeshow chi^2^ was 13.8 (p=0.09).

*Age at 4^th^ examination and propensity score for follow-up was forced into the model.

**Table S3: Odds Ratios (95% CIs) of significant variables in adolescence for hypertension events in adulthood, considering hip circumference as anthropometric variable**

| **Independent variable** | **OR (95% CI)** | **P-value** |
| --- | --- | --- |
| **Model 1** |  |  |
| Age at 4^th^ exam*, years | 1.01(0.90-1.13) | 0.88 |
| Gender, female | 0.14(0.07-0.28) | 0.001 |
| Systolic blood pressure, cm Hg | 1.03(1.00-1.06) | 0.04 |
| Diastolic blood pressure, cm Hg | 1.04(1.01-1.08) | 0.02 |
| Hip circumference, cm | 1.05(1.02-1.08) | 0.001 |
| Propensity score*, % | 1.01(0.99-1.03) | 0.16 |

A forward stepwise approach was considered to keep significant covariates among gender, waist circumference, FBS, TG, HDL-C, TC, eGFR, SBP and DBP with a p-value of <0.2 for enter and >0.05 for removal.

The AUC of the model was 0.84 (95% CI: 0.79-0.89) and its Hosmer-Lemeshow chi^2^ was 17.9 (p=0.02).

*Age at 4^th^ examination and propensity score for follow-up was forced into the model.
